# Association between Mild Cognitive Impairment and Gut Microbiota in Elderly Korean Patients

**DOI:** 10.4014/jmb.2305.05009

**Published:** 2023-07-20

**Authors:** Eun-Ju Kim, Jae-Seong Kim, Seong-Eun Park, Seung-Ho Seo, Kwang-Moon Cho, Sun Jae Kwon, Mee-Hyun Lee, Jae-Hong Kim, Hong-Seok Son

**Affiliations:** 1 Department of Biotechnology, College of Life Sciences and Biotechnology, Korea University, Seoul 02841, Republic of Korea; 2 Sonlab Inc., Seoul 02841, Republic of Korea; 3 AccuGene Inc., Incheon 21999, Republic of Korea; 4 College of Korean Medicine, Dongshin University, Naju 58245, Republic of Korea; 5 Department of Acupuncture and Moxibustion Medicine, College of Korean Medicine, Dongshin University, Naju 58245, Republic of Korea

**Keywords:** Mild cognitive impairment, gut microbiota, gut-brain axis, race, Korean

## Abstract

Recent studies have confirmed that gut microbiota differs according to race or country in many diseases, including mild cognitive impairment (MCI) and Alzheimer’s disease. However, no study has analyzed the characteristics of Korean MCI patients. This study was performed to observe the association between gut microbiota and MCI in the Korean elderly and to identify potential markers for Korean MCI patients. For this purpose, we collected fecal samples from Korean subjects who were divided into an MCI group (*n* = 40) and control group (*n* = 40) for 16S rRNA gene amplicon sequencing. Although no significant difference was observed in the overall microbial community profile, the relative abundance of several genera, including *Bacteroides*, *Prevotella*, and *Akkermansia*, showed significant differences between the two groups. In addition, the relative abundance of *Prevotella* was negatively correlated with that of *Bacteroides* (r = 0.733). This study may provide Korean-specific basic data for comparing the characteristics of the gut microbiota between Korean and non-Korean MCI patients.

## Introduction

Mild cognitive impairment (MCI) is considered an intermediate stage between the cognitive changes of aging and fully developed symptoms of dementia, such as those seen in Alzheimer’s disease (AD) [1]. MCI is associated with a greater decline in cognitive function than normal aging; however, it does not significantly interfere with daily life, making it difficult to diagnose in the early stages. Although many patients with MCI experience memory impairment, their medical examination results are often normal. Some studies have shown that patients with MCI progress to AD at a rate of 10–15% per year, and 80% of these patients are diagnosed with AD approximately after 6 years of follow-up [2]. Therefore, the identification and recognition of MCI is an important issue and challenge to be overcome.

Over the past decades, the role of gut microbiota has been observed primarily in immunological, nutritional, and metabolic functions of the host [3-5]. Recently, the gut microbiota has been reportedly linked to brain processes and behaviors [6]. The link between the brain and gut microbiota is called the Gut– Brain axis. Gut microbiota has been reported to modulate and influence cognitive dysfunction as well as the progression of neurodegenerative diseases. Dysbiosis in the human gut can negatively affect brain function by causing neuroinflammation [7]. For example, recent studies have demonstrated associations between gut microbiota and dementia [8]. Several reports suggest that patients with AD have quantitative and qualitative changes in their gut microbiota [9].

Alterations in the composition of the human gut microbiota are not only determined by health conditions but also by gender, nationality, and ethnicity [10-12]. In addition, some studies on microbiota composition in different ethnic and racial groups have confirmed that the gut microbiota differs according to race in many diseases, such as autism, cancer, irritable bowel syndrome, and obesity [13-19]. Various studies have investigated the association between gut microbiota and MCI by race (*e.g.*, Japanese and Chinese) [8, 20, 21]. These results can enable racial classification of MCI patients and may help to establish recommendations for race-specific treatment methods based on gut microbiota characteristics. However, no study has examined the relationship between the gut microbiota and MCI in the Korean population.

Therefore, the purpose of this study was to examine the association between gut microbiota and MCI in elderly Koreans and identify potential markers for Korean MCI patients. Additionally, potential correlations between the relative abundance of different gut microbiota at the phylum and genus levels were analyzed.

## Materials and Methods

### Study Design

A cohort of elderly Koreans living in community (aged 57–80 years of both sexes) was recruited from the Dongshin University Medical Center (Republic of Korea). Participants voluntarily applied to be subjects for this study and informed consent was obtained from all subjects prior to participation, following the IRB-approved consent form. The medical ethics committee of Dongshin University Medical Center approved the study protocol (DSGOH-2021-004). Neuropsychological tests (Global Deterioration Scale (GDS), Korean Mini-Mental State Examination (K-MMSE), and Korean version of the Montreal Cognitive Assessment (MoCA-K)) were performed to classify the participants into the MCI and control groups. The diagnostic criteria for MCI were as follows: (1) second or third stage of GDS, (2) K-MMSE score ≤ 23, and (3) MoCA-K score ≤ 22. Those who fulfilled two or more of these three criteria were assigned to the MCI group and the rest were assigned to the control group. There was a total of 40 subjects in the MCI group (*n* = 40) and 40 subjects in the control group (*n* = 40). The characteristics of the MCI and control groups are shown in Table 1.

### Fecal Sample Collection and DNA Extraction

Fresh fecal samples were collected in NBgene tubes (Noble Biosciences) and stored at –80°C until analysis. Fecal DNA was extracted using an AccuFAST automation system (AccuGene Inc., Republic of Korea) according to the manufacturer’s instructions. For MiSeq sequencing, bacterial genomic DNA amplification was performed using primers containing 515 forward and 806 reverse bases with Nextera adaptor sequences to target the V4 hypervariable region of the 16S rRNA genes. 16S rRNA genes were amplified through 25 polymerase chain reaction (PCR) cycles using KAPA HiFi HotStart ReadyMix (Roche sequencing, USA). PCR products (~250 bp) were purified using HiAccuBeads for next-generation sequencing (NGS) library purification (AccuGene Inc.). The amplicon libraries were pooled at an equimolar ratio and the pooled libraries were sequenced on an Illumina MiSeq system using the MiSeq Reagent Kit v2 for 500 cycles (Illumina, USA).

### Microbiota Analysis

All reads were denoised by correcting amplicon errors and used to infer exact amplicon sequence variants (ASVs) using DADA2 v1.16 [22]. The SILVA release 138 rRNA reference database [23] was utilized to create a Naïve Bayes classifier to classify the ASVs obtained from DADA2. Downstream analyses of quality- and chimera-filtered reads were performed using the Quantitative Insights Into Microbial Ecology (QIIME2-2022.2) software package [24]. Each sequence obtained from the DADA2 datasets was assigned to taxonomy with a threshold of 99% pairwise identity using QIIME2 workflow scripts and SILVA release 138 rRNA reference database classifier. To explore the metabolic and other functional activities of the gut bacterial communities in control vs. MCI subjects, an open-source bioinformatics tool called phylogenetic investigation of communities by reconstruction of unobserved states (PICRUSt 2) was used. The ASVs generated from the 16S rRNA gene amplicon sequencing data were entered into PICRUSt 2 software. They were analyzed for the prediction of functional genes of the classified members of the gut microbiota resulting from reference-based ASV selection against the SILVA release 138 database. Subsequently, the inferred gene families were annotated against the Kyoto Encyclopedia of Genes and Genomes (KEGG) orthologs and then collapsed into KEGG pathways to generate the functional pathway. Finally, the functions were categorized and compared at levels of 3.

### Data and Statistical Analysis

Bacterial diversity was determined by α-diversity using Chao 1, observed species, Shannon, and Simpson indices) Microbial community clustering (β-diversity) was performed using unweighted and weighted UniFrac distance matrix, Jaccard, and Bray Curtis distance matrices. The linear discriminant analysis (LDA) effect size (LEfSe) method was used to characterize taxa with statistical significance and biological relevance. For LEfSe analysis, the Kruskal–Wallis test (α value of 0.05) and LDA score > 1 were used as thresholds. Pairwise comparisons were analyzed using the Mann–Whitney *U* test. Statistical analyses were performed using GraphPad Prism v.9.3.1 (GraphPad Software, USA). All data are expressed as the mean ± standard deviation. Statistical significance was set at *p* < 0.05. Multiple testing was corrected using the positive false discovery rate (FDR, type 1 error) by computing q-values after the *t*-test. The associations among fecal microorganisms and K-MMSE, MoCA-K, age, and education were assessed using Spearman’s rank correlation analysis.

## Results

### Alpha and Beta Diversity of Gut Microbiota

The analysis of alpha diversity included calculations of Chao 1, observed species, Shannon, and Simpson indices; however, no significant difference was detected between the MCI and control groups (Fig. S1A). Beta diversity was analyzed using Bray Curtis, Jaccard, Unweighted UniFrac, and Weighted UniFrac matrices (Fig. S1B). No significant difference was observed between the two groups in the beta diversity analysis, implying that MCI-related changes might occur only in certain bacterial taxa.

### Taxonomic Distribution and Composition of Gut Microbiota

**Fig. 1:**
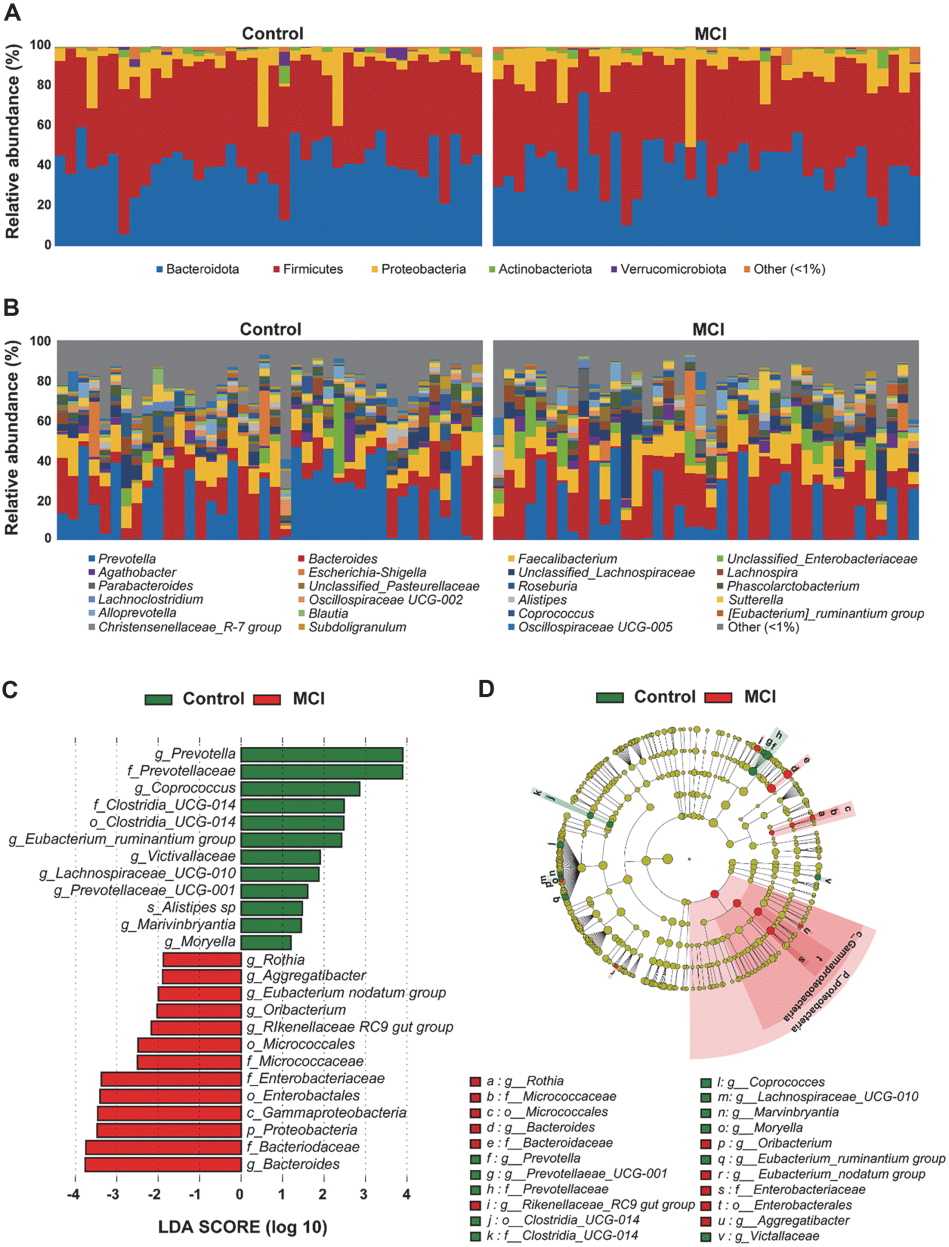
Comparison of the bacterial relative abundance of the control and MCI groups at the (**A**) phylum and (**B**) genus levels. (**C**) Cladogram showing the phylogenetic relationships of bacterial taxa revealed by LEfSe. (**D**) Bar chart showing the log-transformed Linear Discriminant Analysis (LDA) scores of bacterial taxa identified by LEfSe analysis (the logtransformed LDA score of 1.0 as the threshold).

Comparison of the relative abundances of bacterial phylum and genus in the control and MCI groups are shown in Figs. 1A and 1B. At the phylum level, Firmicutes and Bacteroidota were the predominant phyla. In the MCI group, Firmicutes accounted for 46.04% of the total abundance of gut microbiota. The LEfSe and cladogram analysis of the gut microbiota between the MCI and control groups are shown in Figs. 1C and 1D. The genera *Eubacterium nodatum group*, *Oribacterium*, *Rikenellaceae RC9 gut group*, and *Bacteroides* were abundant in the samples from the MCI group, whereas the genera *Prevotella*, *Coprococcus*, and *Eubacterium ruminantium group* were abundant in the samples from the control group.

**Fig. 2:**
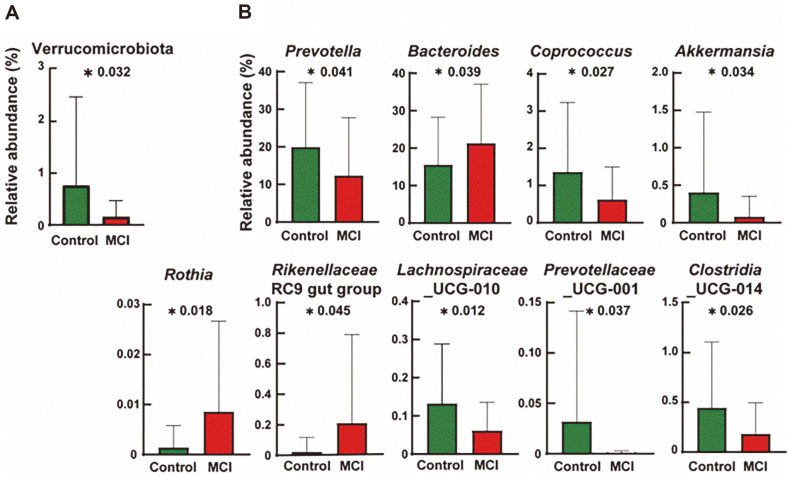
Comparison of the relative abundance showing significant difference between the control and MCI groups at the (A) phylum and (B) genus levels. *p < 0.05.

Microorganisms with significant differences in relative abundance between MCI and the control groups are presented in Fig. 2. The relative abundance of the phylum Verrucomicrobiota in fecal samples was significantly lower in the MCI group than in the control group (Fig. 2A). At the genus level, nine genera exhibiting different relative abundances between the MCI and control groups were identified. (Fig. 2B). The relative abundance of *Prevotella*, *Coprococcus*, *Akkermansia*, *Lachnospiraceae* UCG-010, *Prevotella*ceae UCG-001, and *Clostridia* UCG-014 was significantly lower in the MCI group, whereas that of *Bacteroides*, *Rothia*, and *Rikenellaceae RC9 gut group* was significantly higher in the MCI group.

### Prediction of Changed KEGG Pathways Using PICRUSt Analysis

**Fig. 3:**
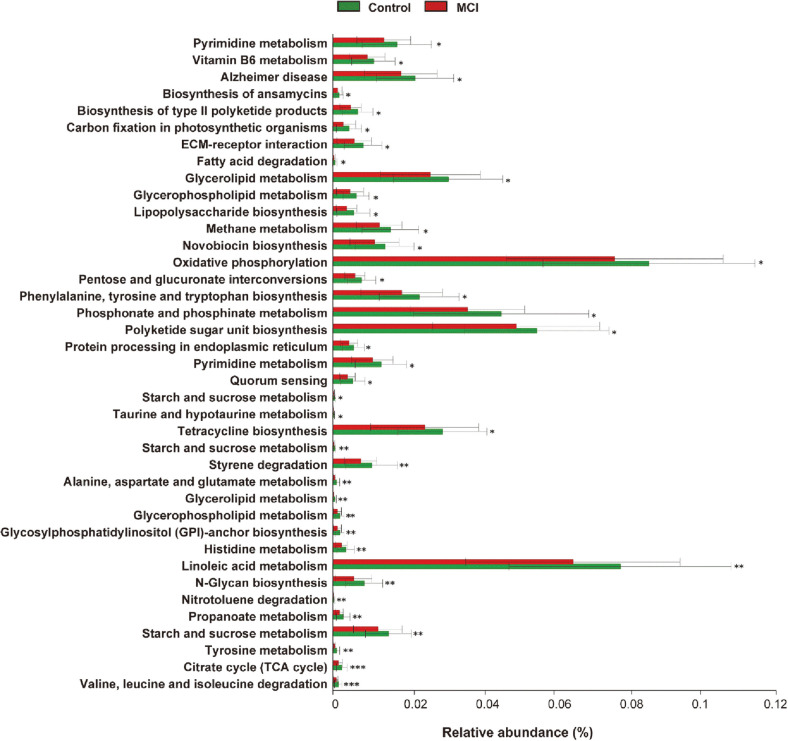
Comparison of the relative abundance of PICRUSt-generated functional profile of gut microbiota. Significant differences in gene categories at level 3 (*t*-test, *p* < 0.05) between the control and MCI groups. Statistical analysis was performed using Student’s *t*-test. *p < 0.05, **p < 0.01, ***p < 0.001.

Functional metabolic pathways related to gut microbiota abundance were determined by PICRUSt analysis using the ASVs table. A total of 10,144 pathways were detected, and 39 primary pathways at level 3 showed statistically significant differences between the MCI and control groups (Fig. 3). The citrate cycle (TCA cycle) and valine, leucine, and isoleucine degradation were found to have the most significant values among the 39 main pathways (*p* < 0.001). In particular, amino acid metabolism (n-glycan biosynthesis; phenylalanine, tyrosine, and tryptophan biosynthesis; taurine and hypotaurine metabolism; and histidine metabolism) was significantly decreased in the MCI group compared to the control group. Remarkably, all 39 primary pathways were markedly higher in the control group than in the MCI group, suggesting that functional gut microbiota may be involved in the pathogenesis and development of MCI.

### Correlation Analysis between Microbiota and Clinical Characteristics

**Fig. 4:**
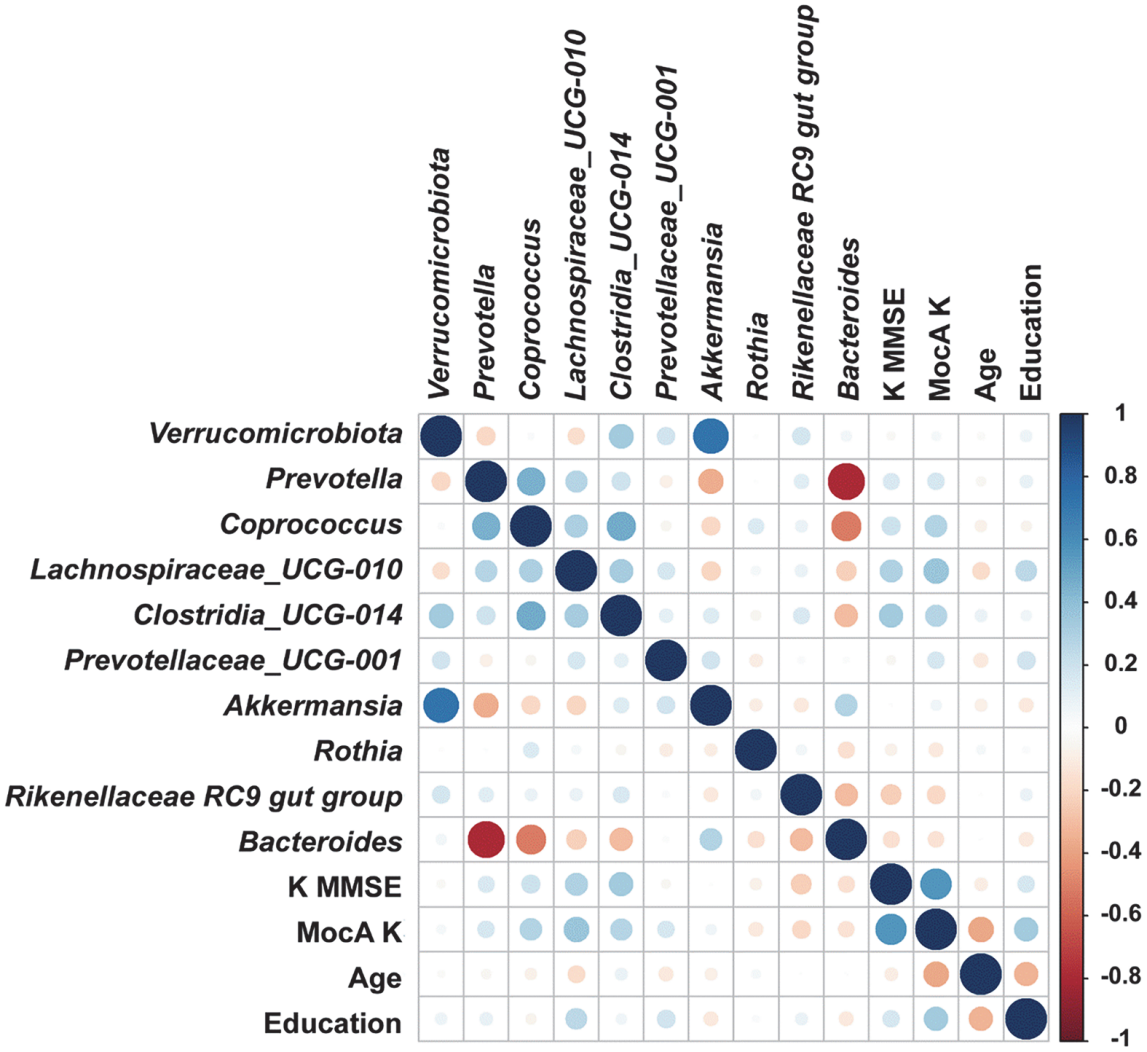
Heatmap of Spearman’s rank correlation coefficients between relative abundance of gut microbiota at the phylum and genus level and clinical characteristics. Circle sizes and color intensity represent the strength of correlation. Blue circles = positive correlations; red circles = negative correlations. Statistical analysis was performed using Student’s *t*-test.

Fig. 4 shows the results of the correlation analysis between the relative abundance of different gut microbiota at the phylum and genus level and the clinical characteristics, indicating evident differences in the MCI and control groups based on the scale scores. The relative abundance of the phylum Verrucomicrobiota positively correlated with that of the genus *Akkermansia* (r = 0.743, *p* value = 0.007). In addition, the relative abundance of genus *Prevotella* was negatively correlated with that of *Bacteroides* (r = 0.733, *p*-value = 0.042).

## Discussion

Many studies analyzing gut microbiota changes in cognitive frailty, AD, and other types of dementia have been conducted using animal and human models. Animal models of cognitive frailty, including AD showed a characteristic composition of the gut microbiota, which was significantly different from that of the control group [25-28]. Observational human studies conducted on fecal samples from patients with different types of cognitive impairment demonstrated changes in gut microbiota in these patients, which could lead to gut microbiota dysbiosis [29-31]. Common clinical conditions of cognitive impairment are associated with dysbiosis of the gut microbiome, which is defined as the loss of number and diversity of microbes symbiotically living in the human gastrointestinal tract [32]. However, in this current study, upon comparing the α- and β-diversity, no significant difference was noticed between the Korean MCI and control groups. These results suggest that the MCI group differed in the relative abundance of some specific microbes, rather than exhibiting a distinct difference in the overall gut microbial profile.

The composition of gut microbiota may vary by race. Chen *et al*. [33] reported that the gut microbial community differed significantly among three races (Asian, American, and European). Furthermore, gut microbiota can vary across regions, even within the same Asian race [34]. Although gut microbiota alterations in patients with different kinds of cognitive disorders, including MCI and AD, have been studied primarily in Asian countries such as Japan and China, no study has analyzed the gut microbiota of the patients with MCI in Korea. Analysis of the gut microbiota of Asian subjects with cognitive dysfunction showed that the genus *Bacteroides* had a higher relative abundance in the MCI group than in the normal group in Japanese subjects, which is consistent with the results of this current study [8]. However, no similar tendency was observed between Chinese and Korean MCI groups. Mingyan *et al*. [21] reported that the relative abundance of *Prevotella* was higher and that of *Bacteroides* was lower in the MCI group than in the normal group among Chinese subjects, which is inconsistent with the results of this current study. These results suggest that the microbes showing significant differences in the MCI group may be different from country to country even within the same Asian population. In addition, the relative abundance of *Prevotella* and *Bacteroides* showed a negative correlation with each other in this study, suggesting that the correlation between gut microbes could be a clue for predicting cognitive dysfunction in Koreans.

The relative abundance of family *Prevotella*ceae, which was significantly lower in the MCI group in this study, was reported to be 77.6% lower in patients with Parkinson’s disease (PD) than in the healthy control group, and this decline might result in reduced mucin synthesis and a subsequent increase in gut permeability [35]. Unger *et al*. [36] reported that patients with PD showed a decrease in the proportion of *Prevotella* in their feces, which might cause a reduction in short-chain fatty acids (SCFAs) [37]. *Prevotella* is also known to be involved in formation of the mucus in the colonic mucosal layer. The reduction in *Prevotella* causes a decrease in intestinal mucus formation and an increase in intestinal permeability, increasing susceptibility to the effects of bacterial antigens and endotoxins [38]. The intestinal epithelium is exposed to large amounts of microorganisms and helminths as well as ingested materials. One of the well-known clinical manifestations of helminth infections is cognitive dysfunction [39]. Mucus is the first line of defense and aids in limiting exposure to all these threats to the epithelium [40]. Therefore, a decrease in *Prevotella* involved in mucus production may contribute to the development of cognitive dysfunction. In this current study, the relative abundance of *Akkermansia* was significantly lower in the MCI group, whereas the relative abundance of *Bacteroides* was significantly higher. *Akkermansia* is considered beneficial to human health and is believed to be a potential probiotic because it can strengthen the epithelial cell layer and modulate the immune system [41]. In addition, *Akkermansia* can produce large amounts of propionate [42]. Propionate has been reported to have multiple beneficial effects on host physiology, such as stimulating gut gluconeogenesis and exerting protective effects in PD patients [43]. The reduction in the abundance of *Akkermansia* has been reported to be associated with dysfunction of the intestinal barrier, which could be an important risk factor for AD [44]. Therefore, it is presumed that the reduced abundance of *Akkermansia* is related to MCI. Meanwhile, the relative abundance of *Bacteroides* was positively correlated with the AD markers p‐tau and p‐tau/Aβ_42_ [45]. Increased abundance of *Bacteroides* may result in increased translocation of lipopolysaccharide from the intestines into systemic circulation and the brain, contributing to AD pathology [46]. Overall, some strains of the genera (*Prevotella*, *Akkermansia*, and *Bacteroides*) in the human gut can regulate immune responses of the intestinal wall through various mechanisms, which are expected to be associated with cognitive dysfunction.

In the current study, the Korean MCI group showed decreased relative abundance of functional metabolic pathways. Among them, orthologs related to amino acid metabolism (n-glycan biosynthesis; phenylalanine, tyrosine, and tryptophan biosynthesis; taurine and hypotaurine metabolism; and histidine metabolism) were significantly lower in the MCI group than in the control group. Lower levels of amino acid metabolism can be associated with decreased neurotransmitter synthesis (*e.g.*, tryptophan can cross the blood–brain barrier and is a precursor of serotonin) [47]. Low levels of amino acid metabolism in the MCI group may contribute to metabolic disorders associated with neurodegenerative disorders and depletion of neurotransmitters, hormones, and immune system modulators. These results not only suggest that gut microbiota may play a role in progressive activation of the immune system but can also contribute to cognitive dysfunction when altered.

Gut microbiome-based therapy is a promising approach for the prevention and treatment of cognitive impairment. However, there are limited clinical studies and many challenges due to differences in genomic sequences, gut microbiota composition, and dietary patterns [48]. Future large-scale studies are needed to clarify the role of gut microbiota in the etiology of cognitive impairment and recommendations for clinical practice. Furthermore, race- and country-specific microbiome data should be accumulated for gut microbiome-based therapies for cognitive impairment to be more effective.

## Conclusion

This study is the first to identify the characteristics of gut microbiota in elderly Korean MCI patients. In this study, although no significant difference was noticed in the whole microbial community profile, the relative abundance of several genera, including *Bacteroides*, *Prevotella*, and *Akkermansia*, showed significant differences between the two groups. The results of the relative abundance of certain genera were consistent with those of MCI patients in other countries; however, certain genera showed different results, suggesting that potential marker microbes in MCI may differ with race. Since this study was conducted with a relatively small sample of the Korean population, it may be difficult to determine potential markers for Korean MCI patients. Nevertheless, this study can provide Korean-specific basic data for comparing the gut microbiota characteristics among Korean and non-Korean MCI groups.

## Supplemental Materials

Supplementary data for this paper are available on-line only at http://jmb.or.kr.

## Figures and Tables

**Table 1:** Baseline characteristics of MCI and control groups.

Characteristics	MCI (*n* = 40)	Control (*n* = 40)
Age (yrs, mean ± SD)	69.5 ± 4.8	66.4 ± 5.8*
Sex (female/male)	34/6	38/2
BMI (kg/m^2^, mean ± SD)	23.9 ± 2.2	23.6 ± 3.0
Education (yrs, mean ± SD)	9.6 ± 2.9	10.9 ± 2.7*
GDS score (mean ± SD)	2.3 ± 0.4	1.0 ± 0.0
K-MMSE score (mean ± SD)	25.7 ± 2.1	28.0 ± 1.3***
MoCA-K score (mean ± SD)	20.0 ± 2.3	25.1 ± 1.7***
Diabetes (%)	47.5	35
Hypertension (%)	25.0	27.5

*p < 0.05, ***p < 0.001 compared to MCI.

## References

[1] Guo M, Peng J, Huang X, Xiao L, Huang F, Zuo Z (2021). Gut microbiome features of chinese patients newly diagnosed with Alzheimer's disease or mild cognitive impairment. J. Alzheimer's Dis.

[2] Lopez OL (2013). Mild cognitive impairment. Continuum (Minneap Minn).

[3] Ejtahed HS, Soroush AR, Angoorani P, Larijani B, Hasani-Ranjbar S (2016). Gut microbiota as a target in the pathogenesis of metabolic disorders: a new approach to novel therapeutic agents. Horm. Metab. Res..

[4] Ejtahed HS, Angoorani P, Hasani-Rabjbar S, Siadat SD, Ggasemi N, Larijani B (2018). Adaptation of human gut microbiota to bariatric surgeries in morbidly obese patients: a systematic review. Microb. Pathog..

[5] Ejtahed HS, Angoorani P, Soroush AR, Ahmad R, Siadat SD, Shirzad N, Hasani RS (2020). Our little friends with big roles: alterations of the gut microbiota in thyroid disorders. Endocr. Metab. Immune. Disord. Drug Targets..

[6] Barrio C, S AS, I MM (2021). The gut microbiota-brain axis, psychobiotics and its influence on brain and behaviour: a systematic review. Psychoneuroendocrinology.

[7] Alsegiani AS, Shah ZA (2022). The influence of gut microbiota alteration on age-related neuroinflammation and cognitive decline. Neural. Regen. Res..

[8] Saji N, Murotani K, Hisada T, Tsuduki T, Sugimoto T, Kimura A (2019). The relationship between the gut microbiome and mild cognitive impairment in patients without dementia: a cross-sectional study conducted in Japan. Sci. Rep..

[9] Jiang J, Liu H, Wang Z, Tian H, Wang S, Yang J (2021). Electroacupuncture could balance the gut microbiota and improve the learning and memory abilities of Alzheimer's disease animal model. PLoS One.

[10] Byrd DA, Carson TL, Williams F, Vogtmann E (2020). Elucidating the role of the gastrointestinal microbiota in racial and ethnic health disparities. Genome Biol..

[11] Schnorr SL, Candela M, Rampelli S, Centanni M, Consolandi C, Basaglia G (2014). Gut microbiome of the Hadza huntergatherers. Nat. Commun..

[12] Dwiyanto J, Hussain MH, Reidpath D, O KS, Oasim A, Lee SWH (2021). Ethnicity influences the gut microbiota of individuals sharing a geographical location: a cross-sectional study from a middle-income country. Sci. Rep..

[13] Gupta VK, S Paul, C D (2017). Geography, ethnicity or subsistence-specific variations in human microbiome composition and diversity. Front. Microbiol..

[14] Brooks AW, Priya S, Blekhman R, Bordenstein SR (2018). Gut microbiota diversity across ethnicities in the United States. PLoS Biol..

[15] Goyal S, Farhana L, Antaki F, Judd S, Nangia MP, Hadden T (2016). Colorectal cancer and racial disparity: gut microbiome as a potential regulator 138. Am. J. Gastroenterol. Suppl..

[16] Carson TL, Wang F, Cui X, Jackson BE, Van Der Pol W, Lefkowitz EJ (2018). Associations between race, perceived psychological stress, and the gut microbiota in a sample of generally healthy black and white women: a pilot study on the role of race and perceived psychological stress. Psychosom. Med..

[17] Pulikkan JP, Maji A, Dhakan DB, Saxena R, Mohan B, Anto MM (2018). Gut microbial dysbiosis in Indian children with autism spectrum disorders. Microb. Ecol..

[18] Yang Y, Zheng W, Cai Q, Shrubsole MJ, Pei Z, Brucker R (2019). Racial differences in the oral microbiome: data from low-income populations of African ancestry and European ancestry. Msystems.

[19] Verhaar BJ, Collard D, Prodan A, Levels JHM, Zwinderman AH, Backhed F (2020). Associations between gut microbiota, faecal short-chain fatty acids, and blood pressure across ethnic groups: the HELIUS study. Eur Heart J..

[20] Liu P, Wu L, Peng G, Han Y, Tang R, Ge J (2019). Altered microbiomes distinguish Alzheimer's disease from amnestic mild cognitive impairment and health in a Chinese cohort. Brain Behav. Immun..

[21] Guo M, Jun P, Xiaoyan H, Lingjun X, Fenyan H, Zhiyi Z (2021). Gut microbiome features of Chinese patients newly diagnosed with Alzheimer's disease or mild cognitive impairment. J. Alzheimer's Dis..

[22] Callahan BJ, McMurdie PJ, Rosen MJ, Han AW, Johnson AJA, Holmes SP (2016). DADA2: high-resolution sample inference from Illumina amplicon data. Nat. Methods.

[23] Glöckner FO, Yilmaz P, Quast C, Gerken J, Beccati A, Ciuprina A (2017). 25 years of serving the community with ribosomal RNA gene reference databases and tools. J. Biotechnol..

[24] Bolyen E, Rideout JR, Dillon MR, Bokulich NA, Abnet CC, Ghalith GA (2019). Reproducible, interactive, scalable and extensible microbiome data science using QIIME 2. Nat. Biotechnol..

[25] Shen L, Liu L, Hong-Fang (2017). Alzheimer's disease histological and behavioral manifestations in transgenic mice correlate with specific gut microbiome state. J. Alzheimer's Dis..

[26] Peng W, Yi P, Yang JJ, Xu PP, Wang Y, Zhang Y (2018). Association of gut microbiota composition and function with a senescenceaccelerated mouse model of Alzheimer's Disease using 16S rRNA gene and metagenomic sequencing analysis. Aging (Albany NY).

[27] Zhan G, Yang N, Li S, Huang N, Fang X, Zhang J (2018). Abnormal gut microbiota composition contributes to cognitive dysfunction in SAMP8 mice. Aging (Albany NY).

[28] Kong Y, B Jiang, X Luo (2018). Gut microbiota influences Alzheimer's disease pathogenesis by regulating acetate in Drosophila model. Future Microbiol..

[29] Barichella M, Severgnini M, Cilia R, Cassani E, Bolliri C, Caroni S (2019). Unraveling gut microbiota in Parkinson's disease and atypical parkinsonism. Mov. Disord..

[30] Ren T, Gao Y, Qio Y, hiang S, Zhang Q, Zhang J (2020). Gut microbiota altered in mild cognitive impairment compared with normal cognition in sporadic Parkinson's disease. Front. Neurol..

[31] Vogt NM, Kerby RL, Dill-McFarland KA, harding SJ, Merluzzi AP, Johnson SC (2017). Gut microbiome alterations in Alzheimer's disease. Sci. Rep..

[32] Sun Y, Baptisa LC, Roberts LM, Jumbo-Lucioni P, McMahon LL, Buford TW (2020). The gut microbiome as a therapeutic target for cognitive impairment. J. Gerontol..

[33] Chen L, Zhang YH, Huang T, Cai YD (2016). Gene expression profiling gut microbiota in different races of humans. Sci. Rep..

[34] Nakayama J, Watanabe K, Jiang J, Matsuda K, Chao SH, Haryono P (2015). Diversity in gut bacterial community of school-age children in Asia. Sci. Rep..

[35] Scheperjans F, Aho V, Pereira PA, Koskinen K, Paulun L, Pekkonen E (2015). Gut microbiota are related to Parkinson's disease and clinical phenotype. Mov. Disord..

[36] Unger MM, Spiegel J, Dillmann KU, Grundmann D, Philippeit H, Burmman H (2016). Short chain fatty acids and gut microbiota differ between patients with Parkinson's disease and age-matched controls. Parkinsonism Relat Disord..

[37] Zhu X, Han Y, Du J, Liu R, Jin K, Yi W (2017). Microbiota-gut-brain axis and the central nervous system. Oncotarget..

[38] Bello-Medina PC, Corona-Cervantes K, Torres NGZ, Gonzalez A, Perez-Morales M, Gonzalez-Franco DA (2022). Chronicantibiotics induced gut microbiota dysbiosis rescues memory impairment and reduces β-Amyloid Aggregation in a Preclinical Alzheimer's Disease Model. Int. J. Mol. Sci..

[39] Romano S, Savva GM, Bedarf JR, Charles IG, Hildebradn F, Narbad A (2021). Meta-analysis of the Parkinson's disease gut microbiome suggests alterations linked to intestinal inflammation. NPJ Parkinsons Dis..

[40] Su J, Braat H, Peppelenbosch MP (2021). Gut microbiota-derived propionate production may explain beneficial effects of intermittent fasting in experimental colitis. J. Crohn's Colitis..

[41] Hou YF, Shan C, Zhuang SY, Zhuang QQ, Ghosh A, Zhu KC (2021). Gut microbiota-derived propionate mediates the neuroprotective effect of osteocalcin in a mouse model of Parkinson's disease. Microbiome.

[42] Ou Z, Deng L, Lu Z, Wu F, Liu W, Huang D, Peng Y (2020). Protective effects of *Akkermansia* muciniphila on cognitive deficits and amyloid pathology in a mouse model of Alzheimer's disease. Nutr. Diabetes.

[43] Jia W, Rajani C, Kaddurah-Daouk R, Li H (2020). Expert insights: the potential role of the gut microbiome‐bile acid‐brain axis in the development and progression of Alzheimer's disease and hepatic encephalopathy. Med. Res. Rev..

[44] Li Z, Zhu H, Zhang L, Qin C (2018). The intestinal microbiome and Alzheimer's disease: a review. AMEM..

[45] Glenn JM, Madero EN, T. Bott NT (2019). Dietary protein and amino acid intake: links to the maintenance of cognitive health. Nutrients.

[46] Angoorani P, Ejtahed HS, Siadat SD, Sharifi F, Larijani B (2022). Is there any link between cognitive impairment and gut microbiota? A systematic review. Gerontology.

