# Supplementary material for: Association between Mild Cognitive Impairment and Gut Microbiota in Elderly Korean Patients
Source: J Microbiol Biotechnol. 2023 Jul 20;33(10):1376–83. doi: 10.4014/jmb.2305.05009 (PMC10619554; doi:10.4014/jmb.2305.05009)
Supplement: Supplementary file 1 [file jmb-33-10-1376-supple.pdf]

J. Microbiol. Biotechnol.
https://doi.org/10.4014/jmb.2305.05009

Supplementary Figure

Association between Mild Cognitive Impairment and Gut Microbiota in Elderly Korean Patients

Eun-Ju Kim[a,†], Jae-Seong Kim[a,†], Seong-Eun Park[a], Seung-Ho Seo[b], Kwang-Moon Cho[c], Sun Jae Kwon[c], Mee-Hyun Lee[d], Jae-Hong Kim[e,*], and Hong-Seok Son[a,*]

[*] Corresponding authors

Jae-Hong Kim (Tel: +82-62-350-7209, E-mail: nahonga@hanmail.net)

Hong-Seok Son (Tel: +82-2-3290-3053, E-mail: sonhs@korea.ac.kr)

[†] These authors contributed equally to this work.

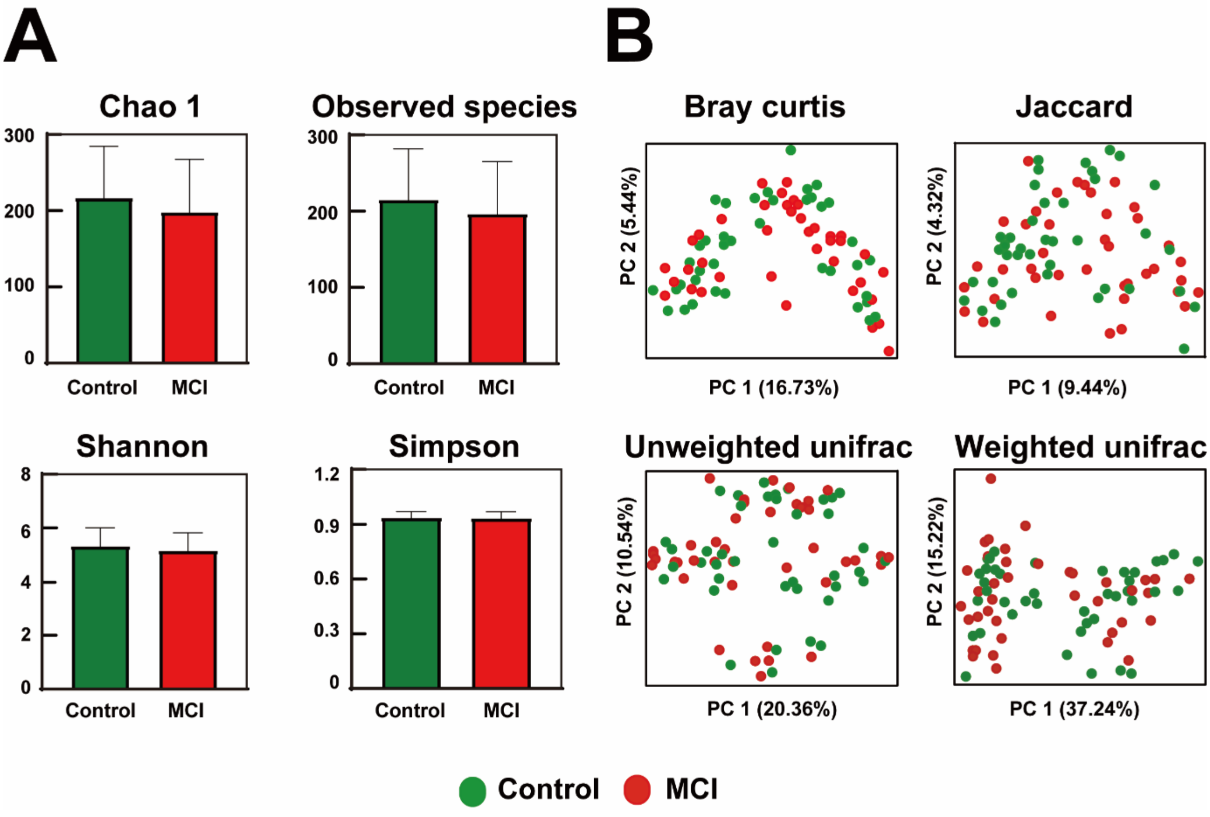

Fig. S1. **(A) Alpha and (B) beta diversity analysis of the gut microbiota in the control and MCI groups.** Alpha diversity of *p*-value is not significant.
